# The Uses of a Year-Book

**Published:** 1906-04-14

**Authors:** 


### THE USES OF A YEAR-BOOK.

A correspondent who has had a large hospital experience in a recent letter urged that it was time somebody dealt with the subject of hospital extension and reconstruction on scientific lines. "Many hospital critics assume that hospital managers have been guilty of extravagance, though they fail to make themselves familiar with the facts and often appear to have little or no knowledge of them." Our correspondent wondered if these critics have ever realised that hospital managers are compelled to spend heavily or their institutions might soon cease to exist under modern conditions of life.

Burdett's "Hospitals and Charities,"* of which the 1906 volume, being the seventeenth year of publication, is now before us, contains ample material for the education of hospital critics. It is much to be regretted that a book of this kind should take so many years to become known, even to those who have most to do with hospital administration. The reason no doubt is that it is regarded more as a directory than a year-book, although in fact it is more of a year-book than a directory. The extent of the information contained in this book may be gathered from the statement that the index of subjects and institutions fills fifty-eight pages, and that any one who will take the trouble to glance through it must be impressed by the exhaustive character of the information, and the thoroughness with which the work has been done. The preliminary chapters contain material which will enable everybody of intelligence to form an accurate judgment of the comparative cost of running a modern hospital, and as to whether or not the institution with which they are identified is extravagantly managed; and if so to what extent and in what direction. The book contains information of the utmost value in reference to the conduct and management of the nursing department. The analysis of the income and expenditure of the various hospitals in the United Kingdom, in India, the Colonies and the United States, all reduced to something like a uniform basis, must prove of great interest, and cannot be found in any other publication.

The uses of a year-book should surely be to provide all who consult it with accurate information upon every portion of the field of work which the book includes. Important as are the financial aspects of the voluntary hospital system, they after all deal only with one branch of work. It is essential however that such a book shall contain in a classified form, so arranged as to make one institution readily comparable with another, details concerning most subjects which affect the thorough administration of the institutions dealt with. Burdett's "Hospitals and Charities" will be found, when tested, to fulfil these conditions with a completeness which it has taken years to accomplish. The constitution of each institution, and its system of management, the qualification of its governors, the distribution of the work among the governing bodies, the names of the principal officials and medical staff, the strength and regulations of the nursing staff, the number of beds, with the special departments and the number of beds in each, the terms of admission, and the regulations as to tickets and patients' payments, and the special features of each institution dealt with, can all be ascertained, together with a mass of details which cannot fail to prove of the utmost value to members of committees, governors and officials who are frequently called upon to compare the working of one institution with another. We have had many years' experience in the use of this book, and we have often been gratified, since we took the trouble to master the plan upon which it is constructed, to find that it seldom or never happens that we cannot ascertain through its pages the facts necessary to form an accurate judgment in dealing with the numerous questions which crop up in regard to the administration of hospitals and kindred charities in the course of each year.

The book has a deservedly high reputation, and although it finds its way into the majority of hospital board-rooms, it would tend to increase the efficiency of many institutions, and to prevent much unintelligent criticism of hospitals, if those who have an opportunity of coming into contact with individual members of the committees managing the various hospitals and institutions throughout the empire, were to call the attention of these gentlemen to this book in order that they may become acquainted with its contents and learn to use it habitually for their guidance and help. We published last week an interesting article on the Growth of Hospital Expenditure Considered in relation to Greater London, which will be found in this year-book. Many of the introductory chapters are well worthy of close study, and we specially commend those on the King's Fund, Hospitals in the United States, the Nursing Department and its Cost, Cold Storage in Hospitals, and the two chapters on Hospital Finance, one devoted to the income and one to the expenditure of hospitals during the year 1904. It is an interesting fact, which ought to be more widely known, that Burdett's "Hospitals and Charities" during the seventeen years of its publication has brought up to date the information contained in "Hospitals and Asylums of the World," so that any institution or individual who has a complete set of these books has in fact an up to date hospital library. The book is beautifully printed, and its appearance and type do great credit to the printers, the Aberdeen University Press.

* Burdett's "Hospitals and Charities," 1906. By Sir Henry Burdett, K.C.B. London: The Scientific Press, Limited, 28 and 29 Southampton Street, Strand, London, W.C. Price 6s. net.

